# Ruptured Left Cornual Ectopic Pregnancy: A Case Report

**DOI:** 10.7759/cureus.41449

**Published:** 2023-07-06

**Authors:** Brittney T Hoang, Donald W Whitaker

**Affiliations:** 1 Department of Obstetrics and Gynecology, Edward Via College of Osteopathic Medicine Auburn Campus (VCOM-Auburn), Auburn, USA; 2 Department of Obstetrics and Gynecology, Hospital Corporation of America (HCA) Florida Fort Walton-Destin Hospital, Fort Walton Beach, USA

**Keywords:** tubal ectopic pregnancy, corneal resection, interstitial ectopic pregnancy, cornual ectopic pregnancy, ruptured ectopic pregnancy

## Abstract

Cornual ectopic pregnancies are rare with a mortality rate that is significantly higher than that of other ectopic pregnancy types. Due to the cornual region's location on the fallopian tube, rupture of a cornual gestation may lead to massive maternal hemorrhage resulting in hypovolemia and shock. Here, we report a 39-year-old female who presented to Hospital Corporation of America (HCA) Florida Healthcare's emergency department (ED) in a state of hypovolemic shock. She was six weeks pregnant based on an unknown and unsure last menstrual cycle. The diagnosis of a ruptured ectopic pregnancy was suspected based on a positive urine pregnancy test and a pelvic ultrasound that revealed an empty uterus and a copious amount of free fluid within the abdomen. Significant hematoperitoneum and hemodynamic instability required emergent exploratory laparotomy with findings of a ruptured left cornual ectopic pregnancy. A left cornual resection and repair was done with an uneventful postoperative period. With cornual ectopic pregnancies being a rare entity, our case emphasizes the importance of early detection and management to help prevent fatal complications.

## Introduction

In the United States, approximately 1.5% of reported pregnancies are ectopic [1]. An ectopic pregnancy develops when an embryo implants outside of the uterine cavity [2,3]. Ninety-eight percent of ectopic pregnancies implant in the fallopian tube, with 80% specifically in the ampullary segment [1]. Only 2% of all ectopic pregnancies develop in the cornual/interstitial region of the fallopian tube [1]. This region is in the part of the fallopian tube that penetrates the muscular layer of the uterus. It measures approximately 1-2 cm in length and 0.7 cm in width and is supplied by Sampson’s artery, which is connected to the ovarian and uterine arteries [4,5]. This area is highly vascularized and potentiates a higher risk of hemorrhage [3]. Ectopic pregnancies are typically diagnosed during the first trimester of pregnancy and are often not recognized until complications have occurred, such as rupture of the ectopic pregnancy [2,3]. Inherently, this condition places the patients at higher risk of hemorrhagic shock in the absence of early diagnosis and management [3,6].

We present an unusual case of a ruptured left cornual ectopic pregnancy in a six-week gestational patient who presented in a state of hemorrhagic shock. We will also discuss predisposing factors, diagnostic challenges, and utilization of early ultrasonographic imaging for early diagnosis.

## Case presentation

A 39-year-old African American female, G10P6036, presented to the emergency department (ED) with primary concern of severe abdominal pain. The patient was found unresponsive in a hotel bathroom and had been thought to be lying there for two days. She stated that she “passed out” with severe abdominal pain and had a minor amount of vaginal bleeding. The patient denied any syncope, orthostatic symptoms, nausea, and vomiting. She was six weeks pregnant based on an unknown but unsure last menstrual cycle. 

Her medical history was significant for six full-term vaginal deliveries (heaviest weight: 6 lbs 13 oz), one elective abortion, and two first-trimester miscarriages. Gynecological history was negative for pelvic inflammatory disease, abnormal Pap smears, or herpes simplex virus. She smokes tobacco, consumes alcohol occasionally, and readily admits to intravenous (IV) drug use. The patient has a known anaphylactic allergy to penicillin. Family history was unremarkable and was negative for breast, uterine, ovarian, or bowel cancer. 

On examination, the patient appeared alert, oriented x4, and responsive and was in a state of severe distress secondary to abdominal pain. The patient's abdomen was distended with positive rebound tenderness and guarding and negative costovertebral angle (CVA) tenderness. Pelvic exam was deferred. Table 1 summarizes the patient's vital signs on initial presentation in the ED. 

**Table 1:** Vital signs on initial presentation in the emergency department.

Vital Sign	Result
Pulse oximetry (%)	99
O_2 _delivery	Room air
Blood pressure (mmHg)	96/67
Temperature (°C)	36.9
Pulse (beats per minute)	123
Respiratory (breaths per minute)	22

Workup included laboratory tests and a summary of the pertinent findings are shown in Table 2. Positive focused assessment with sonography for trauma (FAST) exam revealed diffuse free fluid in the bilateral upper quadrants. Transabdominal and transvaginal ultrasonography were performed. The uterus measured 8.9x4.5 cm with a 3.0 cm cervix. Findings were negative for an intrauterine pregnancy. There was also extensive complex free pelvic fluid, which raised strong suspicion for a ruptured ectopic pregnancy, given the clinical scenario. 

**Table 2:** Pertinent laboratory test findings.

Laboratory test	Result	Reference range
Hematology		
Hemoglobin (Hgb)	8.3	13.7-17.5 gm/dL
Hematocrit (Hct)	25.3	34.1 - 44.9 %PCV
Platelets	313	186-369 K/mm^3^
White blood cell (WBC)	21.67	3.98-10.04 K/mm^3^
Chemistry		
Sodium	133	136-145 mmol/L
Potassium	4.8	3.4-5.1 mmol/L
Blood urea nitrogen (BUN)	27	9-23 mg/dL
Creatinine	1.04	0.55-1.30 mg/dL
Troponin I, high-sensitivity	51.0	0-33 ng/L
Lactic acid	7.4	0.50-2.00 mmol/L
Human chorionic gonadotropin (HCG)		
Urine HCG, qualitative	Positive	N/A
HCG, quantitative	3186.0	0.0-10.0 mIU/mL

The patient was taken to the operating room (OR) without delay. She underwent an emergent exploratory laparotomy under general anesthesia. A Pfannenstiel incision was made and was carried down to the fascia, nicked in the midline, and the fascial incision was extended bilaterally with Mayo scissors. The peritoneum was sharply entered and bluntly opened, the bladder blade was positioned, and the rectus abdominis muscles were taken off the fascia of both the superior and inferior flaps. There was an extensive hematoperitoneum gush from the opening, which was evacuated with suction. A mass was found on the left cornual/interstitial tube, which appeared to be ruptured and actively bleeding. The left ovary, right tube, right ovary, and an anteverted uterus all appeared normal. The patient was placed in the Trendelenburg position, an O'Sullivan-O'Connor self-retaining retractor was placed, and the bladder blade was positioned. The bowel was packed cephalad, and the cuff was grasped with a Babcock clamp including both the cornual region of the uterus and the isthmic portion of the left tube. A short ligature was used with numerous fulguration engagements and then cut; this specimen was sent for pathologic evaluation. A prolonged period of observation was performed, and Arista was placed to assure hemostasis. All packing and self-retaining retractors were removed from the abdominal wall. The fascia was closed with two separate running unlocked 0 Vicryl, and the subcutaneous tissue was copiously irrigated and suctioned. Subcuticular stitches were used to close the skin. The patient tolerated the procedure well and was discharged to the recovery room in a stable condition and extubated.

The patient lost approximately 1300 mL of blood, with less than 10 mL from the operation. Preoperative hemoglobin in the ED was 8.3, and intraoperative hemoglobin was 6.0. She was given two units of packed red blood cells (PRBCs) in the OR. Postoperatively, the patient was followed closely and was given an additional unit of PRBC. Internal medicine consult was obtained to work up the elevated lactic acid, which was thought to be secondary to prolonged immobility and resolved with IV hydration. The remaining postoperative course was uneventful. The patient was discharged home on postoperative day two with Lortab 7.5 #12 and instructed to follow-up with her obstetrician-gynecologist (OB-GYN) in two weeks. 

Of note, the pathology report revealed a left fallopian tube containing numerous chorionic villi, which was consistent with an ectopic pregnancy. The specimen from the cornual resection measured 0.7 cm in length and 1.0 cm in diameter.

## Discussion

Currently, there are no treatments that may prevent an ectopic pregnancy [4]. Predisposing factors include, but are not limited to, inflammation that damages the fallopian tube (e.g., salpingitis and chlamydia), previous tubal ligation, history of infertility independent of tubal disease, history of ovulation induction, prior ectopic pregnancy, prior tubal surgery, smoking, diethylstilbestrol exposure, and advanced age [4,7,8]. Characteristic clinical findings of ectopic pregnancies are based on the classic triad of abdominal or pelvic pain, amenorrhea followed by vaginal bleeding, and a positive human chorionic gonadotropin (b-hCG) [4,8,9].

**Figure 1:**
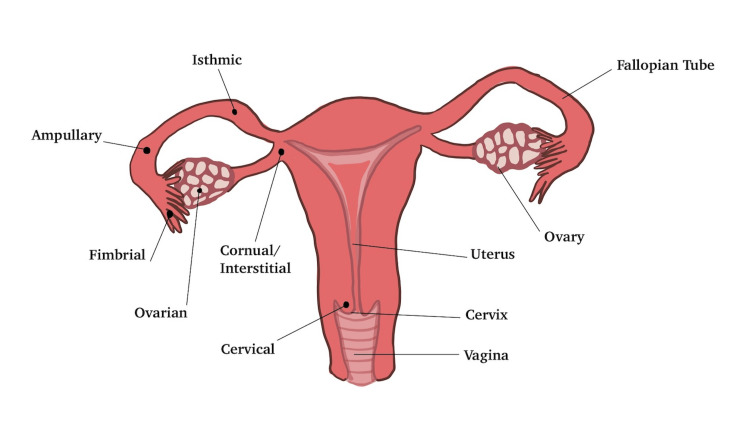
Female internal reproductive organs and typical ectopic pregnancy locations. The right side of the figure identifies the female internal reproductive organs: uterus, cervix, vagina, fallopian tubes, and ovaries. The left side of the figure identifies different types of ectopic pregnancies by location on the female reproductive organs: cornual/interstitial, isthmic, ampullary, fimbrial, ovarian, and cervical ectopic pregnancies.

Cornual ectopic pregnancies have historically been difficult to diagnose due to the cornual region's location and proximity to the uterus. As a result, differentiation of cornual ectopic pregnancies with eccentric uterine pregnancies has been a challenge [4] and may contribute to delayed detection of cornual pregnancies [9]. Patients with cornual pregnancies typically manifest symptoms later in gestation, making the diagnosis of cornual ectopic pregnancies rather difficult [3]. As shown in Figure 1, the cornual region of the fallopian tube penetrates the muscular layer of the uterus. Given the highly vascularized site and extensibility of the uterine wall, rupture of cornual ectopic pregnancies has high risks for massive hemorrhage [9]. Hemorrhagic shock is found in nearly a quarter of the patients, which contributes to the high mortality rate of cornual pregnancies [9]. Consequently, the prevalence of hysterectomy secondary to ruptured cornual pregnancies is estimated at 40%, and there is a 20% risk of uterine rupture if the pregnancy extends beyond 12 weeks of gestation [9,10].

According to the American College of Obstetricians and Gynecologists (ACOG), first-trimester ultrasound exams are not standard as it is too early to visualize the fetus’s limbs and organs in detail [10]. Rather, it is recommended to have a standard ultrasound performed between 18 and 22 weeks of pregnancy when fetal anatomical features are more developed [10]. Early first-trimester ultrasounds are typically done to estimate the gestational age, screen for certain genetic disorders, count the number of fetuses, check the fetus’s heart rate, or evaluate for ectopic pregnancies [10]. A previous study conducted in 1992 by Timor-Tritsch et al. highlighted three diagnostic criteria using ultrasound to help detect a cornual or interstitial pregnancy: an empty uterine cavity, a gestational sac separated from the uterine cavity, and a thin myometrial lining of less than 5 mm around the gestational sac [4,11]. These three criteria have a specificity of 88-93% and a low sensitivity of 40% [11].

Since early first-trimester ultrasounds are not a standard of care, the detection and diagnosis of ectopic pregnancies may go unnoticed until complications have already occurred, as in our patient. With the increasing integration of point-of-care ultrasound by ER physicians and other outpatient providers [3], the identification of cornual pregnancies and utilization of early first-trimester ultrasounds may help improve the rates of diagnosis and prevent life-threatening complications, such as massive hemorrhage [12].

## Conclusions

Cornual ectopic pregnancies continue to be a challenge in terms of diagnosis and management. Early clinical diagnosis supported with ultrasonography may potentially provide conservative management and ultimately reduce mortality. Because diagnosis is typically not made until a rupture has occurred, it is important to recognize common signs and symptoms in an emergency setting to guide clinicians toward an appropriate management plan.
